# Supplementary material for: Chromothripsis during telomere crisis is independent of NHEJ, and consistent with a replicative origin
Source: Genome Res. 2019 May;29(5):737–49. doi: 10.1101/gr.240705.118 (PMC6499312; doi:10.1101/gr.240705.118)
Supplement: Supplemental Material [file supp_gr.240705.118_Supplemental_file_1.zip › contigs/annotated_contigs/DB37/contig.2.DB37_length_735_mean_cov_8.6.docx]

**DB37_length_735_mean_cov_8.6**

```
AAACAAAACACCAAAATGTCTATCTGTAGGAATTTGTTTTCAAGTTGTGATACATAGGTACAGTGAAATATTATACATTCATTTAAAAT
 >chr9:107705353-107705597 - E=6e-132 p=1e-02
GATGATAAAATCTGTATTTGTTTACATGAAAAACTGTCCACTATAATGTAGTGAAAATAATAGATTACAAACAATATATATGGAATAAA

ATTGTTTAGAAACAATTTCTAGAAGAAGGTACAATGGACAGAATTATCTCTGGGAAGTGGGTTT|AT|TTGAAGGAAAGACAAGAAATT
                                                                 >chr9:107696910-107697
TGTTTGTTTTGGATTTACTTGTAGAATGACCTGCAAAATTTTGTCCTAATAAATGTTAACAAGTGGCTTTCCATACAAAAACCAAACCA
397 - E=3e-219
AACAAAACCCTGATGTAAACCAAAAATAAAATTCTGAGGCCCCCCTTCACCATCTGAATGAACTTCCTCCTCTGCAAGGGCACTCTTAA

AATTTAACATGAAAGACTGGTTCAGGTCATGACGGGAAGTGGGGGTCGGACAGGCCTCATTATGCCTCTCTGGCATTAACATCAACACA

GACCTTAAGTCTGATAAGAAGCATTTACAATCTATTCTTTCTGAAGCCTGTTACCTGAAGGCTTCCTCTGCACAATCAGAACTTTGGTT

TCAACAAGAACTTTTCTTAACCCAGACGTTCCTTCCTATTGAACCCAGGTCTTTAGATAAACTCAACCAATTGTCAACCAGAAAAATTT

TTAATTTACCTATAACCT|GGAAACC
```
